# Genome-wide association of rice response to blast fungus identifies loci for robust resistance under high nitrogen

**DOI:** 10.1186/s12870-021-02864-3

**Published:** 2021-02-18

**Authors:** Mathias Frontini, Arnaud Boisnard, Julien Frouin, Malika Ouikene, Jean Benoit Morel, Elsa Ballini

**Affiliations:** 1 BGPI, Univ Montpellier, CIRAD, INRAE, Institut Agro, Montpellier, France; 2 Centre Français du Riz, Arles, France; 3 AGAP, Univ Montpellier, CIRAD, INRAE, Institut Agro, Montpellier, France; 4 Groupe de Valorisation des Produits Agricoles (GVAPRO), Alger, Algeria; 5 BGPI, Univ Montpellier, CIRAD, INRAE, Institut Agro, Montpellier, France

**Keywords:** Rice, *Magnaporthe oryzae*, GWAS, Nitrogen, Robustness, temperate japonica rice, rice blast, induced susceptibility

## Abstract

**Background:**

Nitrogen fertilization is known to increase disease susceptibility, a phenomenon called Nitrogen-Induced Susceptibility (NIS). In rice, this phenomenon has been observed in infections with the blast fungus *Magnaporthe oryzae*. A previous classical genetic study revealed a locus (*NIS1*) that enhances susceptibility to rice blast under high nitrogen fertilization. In order to further address the underlying genetics of plasticity in susceptibility to rice blast after fertilization, we analyzed NIS under greenhouse-controlled conditions in a panel of 139 temperate japonica rice strains. A genome-wide association analysis was conducted to identify loci potentially involved in NIS by comparing susceptibility loci identified under high and low nitrogen conditions, an approach allowing for the identification of loci validated across different nitrogen environments. We also used a novel NIS Index to identify loci potentially contributing to plasticity in susceptibility under different nitrogen fertilization regimes.

**Results:**

A global NIS effect was observed in the population, with the density of lesions increasing by 8%, on average, under high nitrogen fertilization. Three new QTL, other than *NIS1,* were identified. A rare allele of the *RRobN1* locus on chromosome 6 provides robust resistance in high and low nitrogen environments. A frequent allele of the *NIS2* locus, on chromosome 5, exacerbates blast susceptibility under the high nitrogen condition. Finally, an allele of *NIS3*, on chromosome 10, buffers the increase of susceptibility arising from nitrogen fertilization but increases global levels of susceptibility. This allele is almost fixed in temperate japonicas, as a probable consequence of genetic hitchhiking with a locus involved in cold stress adaptation.

**Conclusions:**

Our results extend to an entire rice subspecies the initial finding that nitrogen increases rice blast susceptibility. We demonstrate the usefulness of estimating plasticity for the identification of novel loci involved in the response of rice to the blast fungus under different nitrogen regimes.

**Supplementary Information:**

The online version contains supplementary material available at 10.1186/s12870-021-02864-3.

## Background

Rice (*Oryza sativa*) grown in Europe occupies 450,000 ha with an average yield of 6.5 t/ha [1]. Seventy-five percent of the varieties grown in Europe are derived from the temperate japonica subspecies, that is adapted to short cropping season and low temperatures. In order to improve elite varieties for different traits, a European Rice Germplasm Collection (ERGC) was established and characterized [2]. Among targeted traits, the resistance to rice blast disease is one of the most studied. Rice blast is caused by the fungus *Magnaporthe oryzae* (syn. *Pyricularia* oryzae) which is the main rice disease in France and worldwide [1, 3]. The disease causes a decrease in photosynthetic activity, poor circulation of nutrients, and can lead to leaf death [4]. Fungicide application is one of the main management methods [5].

As part of the Green Revolution, breeding programs have established varieties with complete resistance to *M. oryzae* [5]. So far, more than 100 major resistance loci and 500 Quantitative Trait Loci (QTL) have been identified, with 35 resistance genes cloned [6, 7]. The application of genome-wide association studies (GWAS) has increased our knowledge on the genetics of rice blast resistance [8–18]. However, temperate japonica remains the subspecies with the highest level of susceptibility [19] and the integration of resistance in breeding programs is still needed [11]. Moreover, virulent fungal strains regularly appear two to six years after adopting resistant varieties, and complete resistance is often not durable [20, 21]. For these reasons, partial (quantitative) resistance, which reduces susceptibility levels, appears to be a complementary option [20]. However, partial resistance is particularly sensitive to management practices [3, 22, 23]. In rice, high levels of nitrogen input are conducive to disease development, a phenomenon called Nitrogen-Induced Susceptibility (NIS) [24, 25]. NIS is characterized by an increase in lesion number in varieties displaying partial resistance/susceptibility, whereas complete resistance seems to be robust to nitrogen input [24, 26]. We have previously proposed a model in which plants under elevated nitrogen fertilization show enhanced induction of plant defenses that is then overcome by an increased expression of the fungal pathogenicity program, thus leading to greater susceptibility [26]. In order to deal with this detrimental phenomenon, it is necessary to identify robust forms of resistance unaffected by nitrogen levels or to identify genetic factors controlling NIS. We have previously identified an allele from the Aus Kasalath variety at the *NIS1* locus on chromosome 1 that dominantly confers enhanced susceptibility to rice blast under high nitrogen fertilization, when introgressed into the temperate japonica Nipponbare variety [24]. Despite the identification of *NIS1*, the genetics of NIS remains poorly understood in rice and other plants.

Phenotypic plasticity is defined as a genotype displaying different phenotypes in different environments, while its opposite, robustness, is the phenomena whereby a given phenotype is expressed similarly across environments. Robustness in the presence of specific disturbances is widely studied [27, 28]. Robustness is measured by comparing the mean phenotype between two environments or by measuring the variance of the phenotype between two environments [28]. A first approach involves the building a model based on large-scale multi-environment phenotyping in order to quantify Genome x Environment effects [29, 30]. However, the disadvantage of this approach arises from difficulties in finding reliable models that correlate traits and their environment drivers [29]. For pathogen resistance, a second approach could be to investigate the impact of environmental disturbance on immune traits using genetic approaches [31–33]. Most of the time the strategy used by breeders is to estimate robustness of resistance by evaluating resistance in multi-environment field trials [13, 34] thus limiting plasticity, or to manage the trials with specific stresses [25]. These methods are efficient to identify robust resistance, but do not allow the identification of loci that could contribute to robustness/plasticity without conferring resistance per se. While the molecular mechanisms by which the plant prioritizes resistance in some environments is beginning to be understood [35], the identification of more loci that buffer the impact of the environment on resistance is required to increase our knowledge of NIS.

Indeed, few genetic studies have identified robust disease resistance loci in rice [36, 37] or other pathosystems [38–40]. In order to investigate the impact of a given disturbance on resistance, it may be fruitful to develop a quantitative metric of robustness [41] that can be associated with the genotype. Many stress tolerance indexes were developed by estimating the variation between the stress and control conditions to map the robustness of the yield under drought or salt disturbance [42–44]. These indexes rate phenotypic robustness, genotype-by-genotype. As far as we know, this type of index has not been used in the case of an abiotic disturbance affecting disease resistance.

In this study, we developed an index to determine the robustness of susceptibility to rice blast in the presence or absence of nitrogen fertilization. Our objective was to map resistance loci that are robust in an agronomic environment using nitrogen fertilization that could be useful for breeders in Europe. We used a temperate japonica population grown under two different nitrogen fertilization regimes and identified QTLs by genome-wide association that were either shared or not across the two environments. Using a new index for rice blast resistance robustness, we were able to identify an additional QTL that buffers the impact of nitrogen fertilization on rice blast susceptibility.

## Methods

### Plant material and experimental setup

**Fig. 1:**
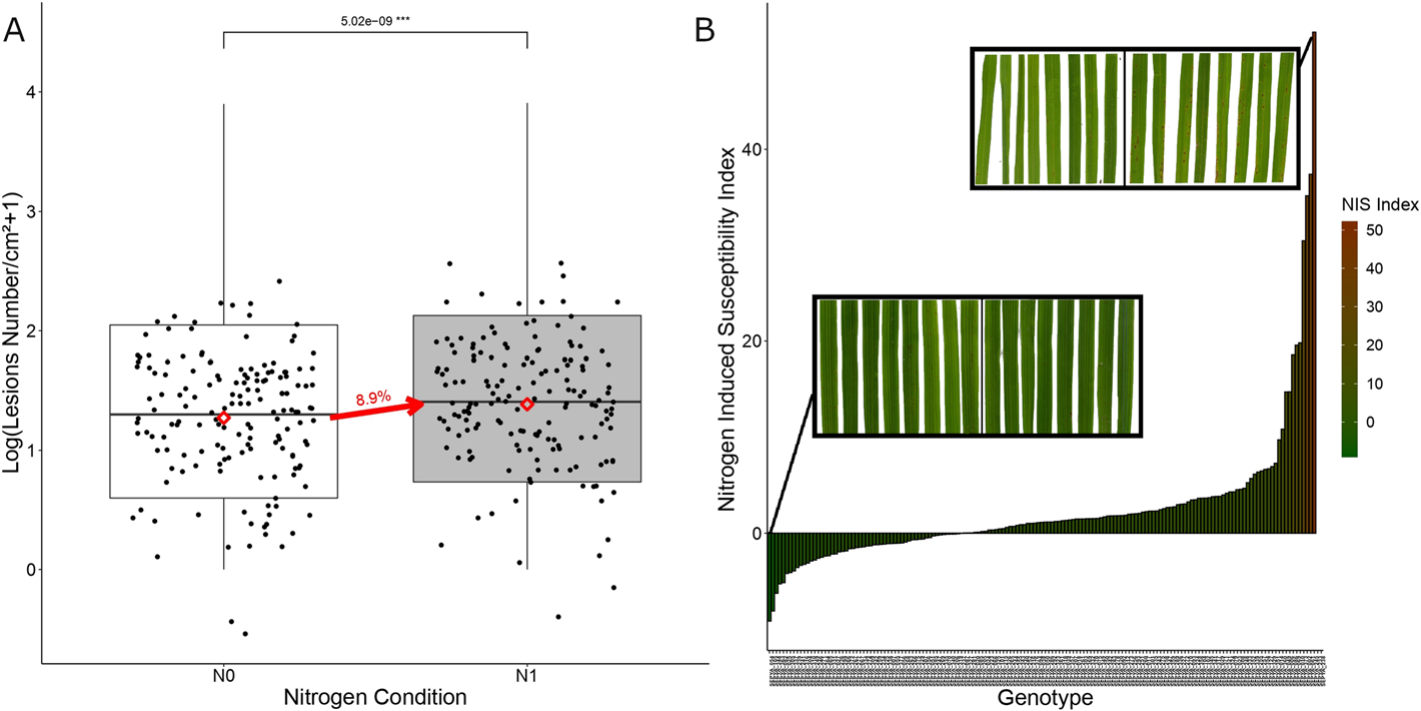
*Disease susceptibility against CD203 isolate in two nitrogen environments.* Each point corresponds to the Lsmean of number of rice blast lesion for one genotype. In white, N0 corresponds to the low nitrogen condition, in grey N1 corresponds to the high nitrogen condition. Red diamonds corresponds to Lsmean for each treatment. The increase of Lsmean between the two conditions is indicated

A set of 331 temperate japonica rice genotypes obtained from the European Rice Germplasm Collection [2] were screened at seedling stage for their susceptibility to *M. oryzae* isolates CD203 and CL26 (Fig. 1 and additional Fig. 4). Among these lines, 159 and 117 demonstrated susceptibility to either CD203 or CL26 respectively, and were selected to investigate partial resistance to *M. oryzae* under different nitrogen fertilization environments (Additional files 1 and 2). The experiment was conducted in greenhouses using previously described experimental procedures [24]. Nineteen replicate blocks of the experiment were conducted in a complete randomized design, with one control genotype, GINES, included in each trial. Two repetitions of four seeds of each accession were sown per plastic plot (9x9x9 cm) in Neuhaus S soil in which poudzolane was added (2 L/ 70 L). Rice plants were grown 28 °C/20 °C, 16 h light. Plants grew under standard fertilization for three weeks. In the fourth week, and one day before inoculation, the N0 (all nutrients except nitrogen) and N1 (nutrients including nitrogen 50% NH4+/50% NO3−; 40 mg/L) treatments were applied as previously described [24]. One statistical unit corresponds to one leaf of one genotype in one condition. The fungal strains CD203 and CL26 were used for inoculation on four-week-old plants (4-leaf stage) with a 50,000 spores/mL suspensions as previously described [45].

### Disease assessment

Seven days after inoculation, each infected leaf was scanned at the same resolution (600 pixels per inch). The pictures were analyzed by LeAFtool (Lesion Area Finding tool), which is an R package developed in-house and available on github depository (https://github.com/sravel/LeAFtool). The program measures lesion number and leaf area. Parameters used for pictures analysis were at least 10,000 pixels for leaves and 50 pixels for lesion areas, with a blur at 3. To account for outliers and software mistakes, aberrant sized lesions have been removed from the analysis. Finally, leaf susceptibility was estimated by the number of a lesion per cm^2^ of leaf area.

The following fixed model has been applied on all data to evaluate genotype, treatment, and interaction effects with the *lme4* R package [46]. Analysis of variance (ANOVA) and Least Square means (LSmeans) calculation was done from this model with the *lsmeans* R package [47]. Data were log-transformed to get parametric distribution.

$$ Y=\mu +{\gamma}_k+{\alpha}_i+{\beta}_j+{\left(\alpha \beta \right)}_{ij}+{\varepsilon}_{ij k} $$

With *Y* as the phenotype, *μ* as the theoretical mean, *γ*_*k*_ the trial effect, *α*_*i*_ the genotype effect, *β*_*j*_ the nitrogen effect, (*αβ*)_*ij*_ the genotype x nitrogen interaction effect, and *ε*_*ijk*_ as residuals. Broad-sense heritability was calculated separately for each Nitrogen environment using analysis of variance, with h^2^ = σ^2^G/(σ^2^G+σ^2^e/n), where σ^2^G and σ^2^e are the estimates of genetic and residual variances.

$$ { NI SI}_i=\frac{1-\frac{LSM_i^{N1}}{LSM_i^{N0}}}{NI} $$

We adapted the previously described Drought Susceptibility Index (DSI) [44], to calculate a Nitrogen Induced Susceptibility Index (NISI):

Where ${LSM}_i^{N0}$ and ${LSM}_i^{N1}$ correspond to the lsmean of genotype *i* in the N0 and N1 conditions respectively. *NI* is the Nitrogen Impact (adapted from the Stress Intensity of the initial DSI) on all genotypes present in the experiment and is calculated as:

$$ NI=1-\frac{LSM_{all}^{N1}}{LSM_{all}^{N0}} $$

NISI, therefore, is a measure of NIS estimated for each genotype relative to the nitrogen impact on the population. In terms of robustness, the index evaluates the observed robustness of disease susceptibility in a given genotype across the two fertilization regimes relative to the total robustness of the panel. If nitrogen induces a global increase in susceptibility to blast, then NI will be negative, and vice versa were nitrogen to reduce susceptibility. When NI is negative, more robust genotypes will have NISI values less than one and approaching zero, indicating that the susceptibility of these genotypes is less affected by nitrogen conditions. Less robust genotypes will have NISI values greater than 1. We use the NISI when there is a statistically significant nitrogen effect on the panel (meaning a NI≠0). In the event no global effect was found, we would use an adjusted NIS index, which corresponds to the same calculation but without the division by NI. The heritability of the NISI has been estimated from the genetic variance observed in the GWA study divided by total variance.

## Genotyping

Genotype data used in this study were obtained from CIRAD [48]. In our panel, we filtered markers with a call rate below 75%, a heterozygosity rate above 20% or a minor allele frequency (MAF) < 2.5. This matrix has been imputed with Beagle 4.0 (window=250 overlap=25 ne=200). The final matrix includes 19,997 SNPs with 331 genotypes.

### Linkage disequilibrium and genome-wide association analysis

We used a genotype matrix, G, of 19,997 SNPs for 331 *japonica temperate* cultivars to calculate Linkage Desiquilibrium (LD), a Kinship matrix, and PCA using TASSEL software 5.2.59 [49]. Linkage Disequilibrium was estimated from the Kinship matrix as the square of the correlation of allelic states (r^2^) between all pairs of markers on each chromosome. The plot of r^2^ values and genetic distance was done with the R package LDheatmap [50]. The Kinship matrix K was estimated by the Centered IBS method as described in [51]. The population structure data were estimated with a Principal Component Analysis (PCA) with 4 axes kept. The GWAS was conducted with a weighted mixed linear model (MLM) on a subset of 139 genotypes from the 159 that were phenotyped. Each GWAS was done with the same G, K, and PCA data, for three phenotypic datasets: LSM^N0^, LSmean of susceptibility in N0 condition for each genotype, LSM^N1^, same with N1 conditions, and the NISI, as calculated using the formula above.

The threshold to declare a significant association was set to –log10 *P* = 5. A quantitative locus was identified if five SNPs were detected with a –log10 P close to 5 in same LD block. Isolated significant SNPs are reported in an additional data file (additional data file 8) but were not further investigated. Gene annotations for selected loci were made using the Nipponbare temperate japonica rice reference genome viewed with the Orygenesdb genome browser [52]. For the most significant QTL, haplotype groups were identified using SNP-seek [53] and then correlated to our genotypic dataset. We ran a new model including the haplotype effect to test the contrast test with a Tukey adjustment to estimate a haplotype group effect. The SNP-seek database was used to identify rice varieties carrying two corresponding haplotypes at *NIS3* locus in order to generate a validation set of twenty-six genotypes from four different subspecies: sixteen with the *NIS3–1* haplotype and ten with the *NIS3–3* haplotype (Table in Additional file 3). This validation panel was inoculated under the same conditions as those used for GWAS (high and low nitrogen followed by inoculation with *M. oryzae* CD203 isolate).

## Results

### NIS is rare, weak, and isolate-dependent in temperate japonica rice genotypes

Partial resistance/susceptibility to rice blast was estimated under two nitrogen fertilization condition levels (low-level: N0; high-level: N1- see Methods) from a least-square means (Lsmeans) analysis of the density of blast lesions. We found 117 *temperate japonicas* (Additional file 3) genotypes that were susceptible to the blast fungus isolate CL26, but no evidence of a global effect nitrogen fertiliser treatment on susceptibility (Additional file 4). There was a significant genotype by nitrogen treatment interaction, with four genotypes (3%) showing an increase of susceptibility under nitrogen treatment and ten (8%) showing a decrease.

We found 159 rice lines susceptible to the blast fungal isolate CD203. In this analysis, the nitrogen fertiliser treatment, genotype, and the genotype-by-treatment interaction were all significant (Additional file 5). The significant nitrogen treatment by genotype interaction implies that the impact of nitrogen on rice blast susceptibility depends on the rice genotype in this panel and thus that these data are suitable for a genome-wide association analysis.

The average increase in susceptibility to CD203 under the N1 condition was 8% (Fig. 1). Nineteen genotypes (11%) were significantly more susceptible in N1 compared to N0 conditions and five were significantly less (3%). The maximum increase in lesion number was observed in the TRAMONTO genotype, with four times more lesions in N1 condition than in N0. Conversely, the MARENY genotype showed three times fewer lesions in N1 condition than in N0 condition. Thus, our results indicate that NIS is relatively rare (14% of the panel) and weak (8% average increase) in this temperate japonica population. Heritability of Lsmeans for rice blast resistance was 0.43 and 0.42 for N0 and N1 conditions respectively (Additional file 6), allowing for GWA analysis within each fertilization condition.

LSmeans of N1 and N0 condition were used for the calculation of a new NIS Index (NISI, see Methods). This index allows the detection of genotypes for which the change of susceptibility upon fertilization deviates from the norm of 8% found at the level of the panel (Fig. 1). A genotype with a NISI of 1 has an increase of susceptibility equal to the global increase (8%). More robust genotypes will have NISI scores approaching zero, while less robust genotypes will have scores greater one. Twenty-seven percent of the genotypes showed a rather robust susceptibility with a NISI between − 1 and 1, and 30 % had a NISI higher than three (Additional file 7).

### GWA analysis of rice blast resistance under different nitrogen fertilization

**Fig. 2:**
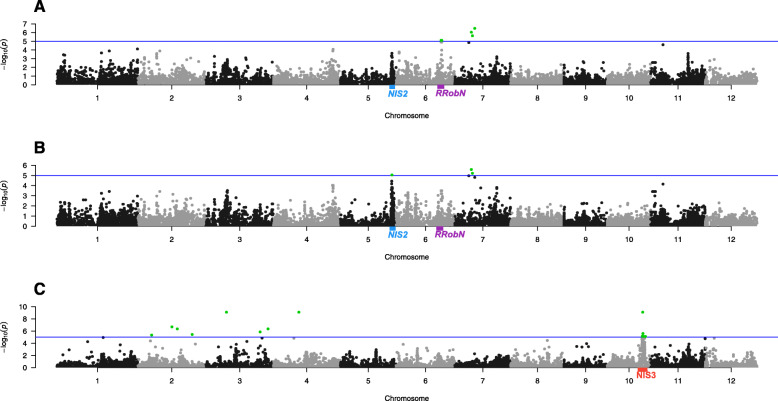
*Manhattan plots from genome-wide association mapping of rice blast su*sceptibility *under contrasted nitrogen fertilization and of the NIS Index.* GWAS result of Lsmean of number of lesions by leaf area in N0 condition (**a**), N1 condition (**b**) and Nitrogen Induced Sensitivity Index (**c**). The Y-axis represents -log10(pvalue) and X-axis indicates the position of the SNP on the chromosome based on the Nipponbare reference genome. The CD203 isolate was used for the inoculations. Green points indicate significant SNPs with -log10(P) ≥ 5

The GWA analysis identified fourteen significant SNPs in the N0 environment and three SNPs in the N1 environment (Fig. 2a and b respectively, and Additional file 8). To define a QTL, we only considered loci detected by several closely linked significant and sub-significant SNPs (see Methods). This excluded five SNPs found on chromosome seven that appeared to be scattered, with no other sub-significant SNP nearby. By contrast, the single SNP found on chromosome 5 appeared to represent a locus (*NIS2*) containing four sub-significant SNPs. Another locus, *RRobN1* on chromosome 6, was defined by eleven significant SNPs.

Twenty-one significant SNPs were identified in the genome-wide association using the NIS Index score as the phenotype (heritability of NISI = 0.1) (Fig. 2c and Additional file 8). Among them, the four SNPs mapping on chromosome 2 and three on chromosome 3 were not considered further as they were scattered all along the chromosomes. A QTL (*NIS3*), defined by a block of 12 significant SNPs, was identified on chromosome 10. It is noteworthy that this QTL was not detected in the GWA analysis for lesion density, further demonstrating the usefulness of the NIS Index.

### RRobN1, a QTL conferring partial resistance not affected by nitrogen fertilization

**Fig. 3:**
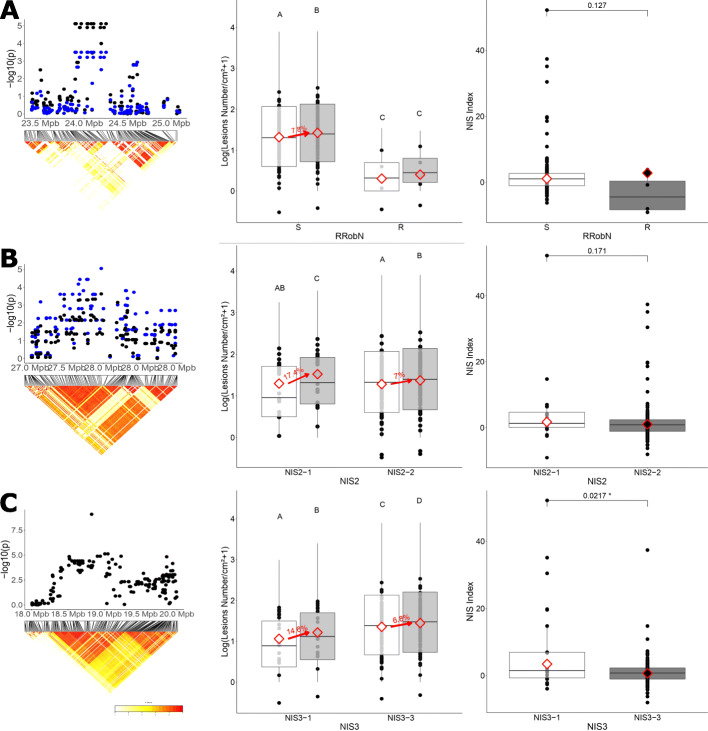
*Genetic details and phenotypic associations of the three loci controlling NIS to M. oryzae in temperate japonicas.* Three loci were identified after GWAS analysis of disease levels against CD203 isolate*: RRobN1* locus (23,9 Mpb − 24,2Mpb on chromosome 6; **a**), *NIS2* locus (27.55–27.96 Mb on chromosome 5; **b**) and *NIS3* locus (18,4 Mpb-19,2Mpb on chromosome 11; **c**). For each locus, the figure on the left represents the physical position of the QTL on their respective chromosome. Heatmaps show the extent of Linkage Desiquilibrium established by R^2^ calculation between each SNPs. The QTL is established for an entire LD block containing significant SNPs. For A and B, the points show the SNPs *p*-value of the GWAS for blast susceptibility. Black dots represent *p*-values obtained in the N0 condition and blue for the N1 condition. For C, the dots show p-values for SNPs identified in the GWAS for the NIS index phenotype. The figures in the middle show susceptibility to CD203 strain for each haplotype of each QTL, in each of the two nitrogen conditions. Each black point corresponds to the Lsmean of the number of rice blast lesions in one genotype. In white, N0 corresponds to the low nitrogen condition, in grey N1 corresponds to the high nitrogen condition. Red diamonds corresponds to the mean Lsmean for each treatment. The red arrow indicates the value of the increased susceptibility in case of significant effect of nitrogen condition. Statistical groups are determined from a pairwise comparison with Tukey adjustment based on a model with Trials and genotypes as covariate. The figures on the right represent the NIS index depending of each QTL haplotype. Although this Index was only used for the identification of *NIS3*, we also displayed it for *RRobN1* and *NIS2* for comparison. Each point corresponds to the NIS Index for each genotypes. Red diamonds correspond to Lsmeans of each haplotype. *P*-Values were obtained from Wilcoxon test for **a** and **b**, and a Student Test after a logarithmic transformation for **c**

The *RRobN1* (Resistance Robust to Nitrogen 1) locus on chromosome 6 was initially identified in the GWA analyses by eleven SNPs significant in the N0 but not the N1 condition, although many of the SNPs at this locus were sub-significant under this condition (Tables Additional files 8 and 9; and Fig. 3a). The haplotype associated with resistance is found in only four varieties of the panel: IRAT 268, IAC 26, GIGANTE VERCELLI, and RUBI. However, an ad hoc analysis considering only this locus suggests that it also conferred resistance under in the N1 environment (Fig. 3a). The level of resistance conferred by this locus was similar to other known major resistance genes [54], with a reduction in lesion number of 75% in both N0 and N1 conditions. However, *RRobN1* does not confer resistance to the CL26 isolate (Additional file 10). Thus *RRobN1* is isolate-specific and confers robust resistance under high nitrogen, two characteristics found for classical major resistance genes [24], suggesting that this locus underlies a classical resistance gene. The *RRoN1* locus (650 kb between 23.90 to 24.2 Mb) on chromosome 6 does not contain any mapped or cloned resistance genes [54] and among the forty-two genes in the region there are no resistance gene analogs in the reference genome Nipponbare (Table Additional file 11). Thus the robust, elevated resistance associated with the *RRobN1* locus may not be conferred by a classical resistance gene analog. Two phospholipidase D genes involved in disease resistance [55, 56] could be good candidates, among others.

### NIS2, a QTL associated with increased susceptibility under high nitrogen fertilization

A second locus on chromosome 5, named *NIS2*, was only detected in the N1 condition against the CD203 isolate. There was no significant association of *NIS2* with the CL26 isolate (Additional file 10). *NIS2* was defined by a unique SNP in a strongly linked LD block (LD r^2^ around 0.9 from 27.55 to 27.96 Mb) containing five sub-significant SNPs (with –log10 P greater than 4) (Fig. 3b). In fact, possibly two haplotypes could be defined at this locus, with the first haplotype (*NIS2–1*) found in 22 varieties (26% of the population). Plants with this *NIS2–1* haplotype showed a 17% increase in lesion number from low to high nitrogen conditions (Fig. 3b). The second haplotype, *NIS2–2,* showed a more modest increase of 7%, similar to the average effect of nitrogen in the population (Fig. 1).

In order to investigate the underlying functional basis of the phenotype conferred by the *NIS2* locus, all 69 genes present at this locus were investigated (Additional file 11). Among the many possible candidates, three functional hypothesis were retained. First, the increase of susceptibility could be due to the presence of regulators of biotic stress responses like *RACK1B* [57], *OsNINJA1* [58, 59] and *OsSYP71* [60]. Second, the phenotype could arise from antagonistic pleiotropy in a gene such as *OsMATE2* [60, 61], that regulates plant growth in response to nitrogen while negatively affecting disease resistance. Third, the phenotype could be due to genes modulating metabolism such as *OsMYB55,* which modulates amino acid metabolism [61], or *OsNADH-GOGAT2* which is involved in nitrogen metabolism [62].

### NIS3, a QTL conferring partial resistance strongly impacted by nitrogen fertilization

In contrast to *RRoN1* and *NIS2*, *NIS3* was identified using the NIS index (NISI; see Methods and above). *NIS3* is located in an LD block of 800 Kb on chromosome 10 (Fig. 3c and Tables Additional files 8 and 9). We could define three haplotypes at this locus using the nineteen SNPs available in the 3000 rice genomes (Table Additional file 13). We could not further characterize the *NIS3–2* haplotype because it was represented by only four genotypes in our panel. The *NIS3–3* haplotype was the most represented in our sample (112 genotypes or 79%) whereas the *NIS3–1* haplotype was less frequent, present in 25 genotypes. The mean NISI value of lines carrying the *NIS3–1* haplotype was 3.46, which was significantly greater than lines with the *NIS3–3* haplotype, which had a mean NISI value close to zero (Fig. 3c). NIS3–1 genotypes were more resistant than NIS3–3 genotypes, but also more sensitive under high nitrogen, with an increase of susceptibility of 14.6% (Fig. 3c).

**Fig. 4:**
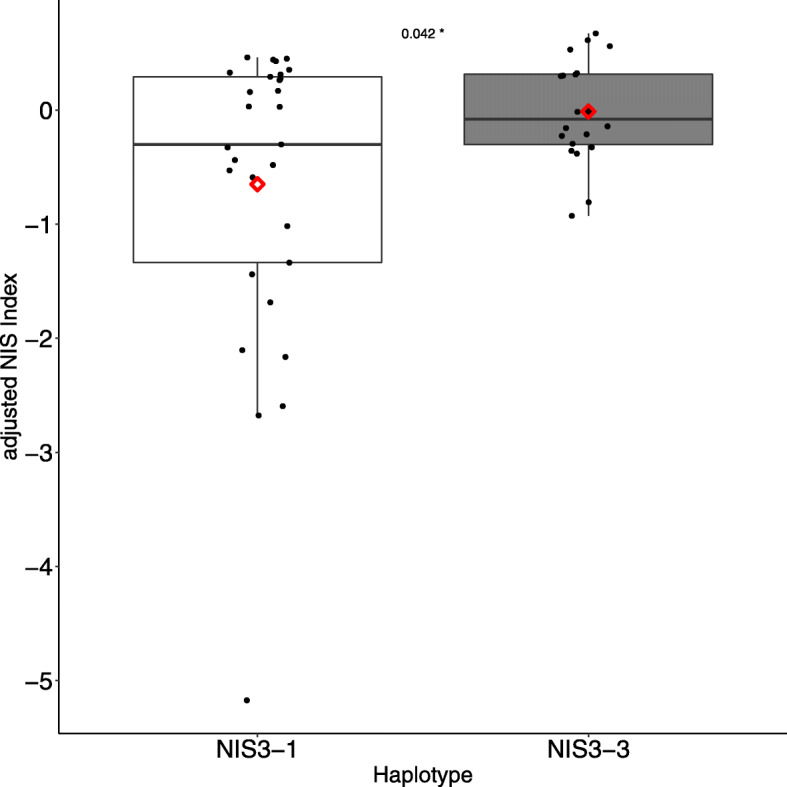
Adjusted NIS index for *NIS3* in validation population*.* Twenty-six genotypes were selected from the 3000 genomes for validation of the QTL based on their *NIS3* haplotype. The CD203 strain was used for inoculation. Each point corresponds to the adjusted *NIS index* of one repetition of one genotype calculated as: $$ 1-\raisebox{1ex}{$ LSmeanN1$}\!\left/ \!\raisebox{-1ex}{$ LSmeanN0$}\right. $$. According to the formula, as values become more negative, there is a stronger effect of nitrogen in increasing susceptibility. Positive values correspond to a decrease of susceptibility. P-Values were obtained from a Student Test

We built a new rice panel (See Methods) in order to specifically test if haplotypes at the *NIS3* loci were predictive of the phenotype across rice diversity. We chose twenty-six genotypes from different subspecies: sixteen with the *NIS3–1* haplotype and ten with the *NIS3–3* haplotype (Table in Additional file 2). This panel was inoculated with the CD203 isolate under the same conditions as those used for GWA. This experiment showed a significant interaction between nitrogen and genotypes (*p*-value=4.33e^− 4^) but no global nitrogen impact (p-value= 0.56). We did not use the NI because there was no global nitrogen effect in the ANOVA (NI~ 0, thus NISI has to be adjusted; see Methods). As we observed in the original GWA, plants with the NIS3–3 haplotype were more robust to the effect of nitrogen treatment, with a mean adjusted NISI score of 0.68, compared to the adjusted NISI score of 3.46 for plants with the NIS3–1 haplotype. These results validate the involvement of *NIS3* in NIS to the CD203 isolate (Fig. 4). There was no significant difference between the adjusted NIS index of haplotypes *NIS3–3* and *NIS3–1* (Additional file 14) in the panel of 117 genotypes susceptible to CL26 strains.

In order to understand the possible genetic basis of the observed increased susceptibility in the high nitrogen environment, the 126 genes annotated at *NIS3* were investigated (Additional file 15). We considered as good candidates genes either involved in biotic stress response (twenty-nine pathogenesis-related genes), abiotic stress response (*OsTIP3;*1, [63] and *RIPER6* [64]), the regulation of both stresses (*OsGRXS17,* [65] and *OsTPR1* [66]), or nitrogen metabolism (*OsPORB,* [67] and a polylpolyglutamate synthetase [68]). The possible modulation of the *M. oryzae* pathogenicity program in response to a high nitrogen environment was also a hypothesis based on our previous results [26]. Interestingly, *OsTPR1* codes for a protein that competitively binds the fungal effector *MoChia1*, allowing the free accumulation of chitin and the re-establishment of the immune response during rice *M. oryzae* interaction [66]. Finally, the NIS response could be due to a buffering of the immune response by primary metabolism at this locus. In that respect, *OsPORB* and the polylpolyglutamate synthetase genes have been shown to be repressed during blast infection [26].

The *NIS3–3* allele is common in temperate japonica (79% of the panel tested, Additional file 16). We searched for interesting allelic variants of genes at the NIS3 locus that could have been fixed in this population. We found that an allele of *RIPER6* (Ripening-related family protein), which is associated with cold tolerance [64], is observed in 90% of the cases in association with *NIS3–3.*

## Discussion

In this study, we demonstrated the negative impact (8% on average) of nitrogen fertilization on rice partial resistance to *M. oryzae* strain CD203. Until now, the Nitrogen-Induced Susceptibility (NIS) phenotype had only been observed on a limited number of rice genotypes [24]. We thus extend the characterization of NIS to an entire rice subspecies. We have also demonstrated that this phenotype varies among genotypes, with a significant Genotype x Nitrogen interaction. We provide evidence that a very small number of genotypes show increased resistance under high nitrogen (Additional file 1), a promising finding for breeders. We also identified a QTL, *RRobN1*, with an allele conferring partial resistance even in the high nitrogen environment. These results indicate that genetic solutions for robust resistance to the blast fungus across nitrogen environments are available in rice temperate japonicas.

Moreover, in addition to the already known locus *NIS1* [24], we identified two QTL (*NIS2* and *NIS3*) implicated in NIS phenotype. Finally, we observed variation in the effects of the QTL depending on the pathogen strain, suggesting that the effect of nitrogen on plant resistance may vary from one fungal isolate interaction to another, like in other pathosystems [69–71].

The change in susceptibility to rice blast under nitrogen fertilisation was twice as large in plants carrying the *NIS2–1* allele than the mean reaction of the panel (a 17% increase instead of 8%, Fig. 3b). Similarly, plants with the *NIS3–1* allele showed a greater increase (15%) in susceptibility following nitrogen treatment than *NIS3–3* genotypes (7% Fig. 3c). In this respect, the *NIS2–1* and *NIS3–3* alleles have similar consequences on susceptibility under high nitrogen. However, *NIS3* differs from *NIS2* because its impact on susceptibility is observed across the nitrogen environments. Indeed, plants with the *NIS3–1* haplotype were observed to be less susceptible than those carrying *NIS3–3* independent of nitrogen treatment, while *NIS2–1* differs from *NIS2–2* only under conditions of high nitrogen.

The use of NIS Index (NISI) proved useful for measuring changes in susceptibility following nitrogen treatment since the *NIS3* locus was only detected in the GWAS using the NISI as our phenotype. Indeed, the NISI allowed us to recognize genotypes both more and less robust to the effects of nitrogen fertilization on susceptibly compared to the mean reaction of all the population. Such an index is particularly useful when a uni-directional effect is observed, as in the case of drought and salinity, which quite often have a similar effects on the whole panel but with varying intensity [43, 72, 73]. Similar indexes were used in other association studies on the robustness of the yield to abiotic stress in various species [44, 74]. Other indexes of robustness that do not take into account an overall effect in the population do exist, and it would be interesting to examine their utility in cases such as NIS [43]. Our study shows that using a robustness index is promising in GWA studies on disease susceptibility induced by perturbations of the environment. However, the results of the CL26 experiment calls for caution. Contrary to the global and unidirectional effects on yield of the abiotic stresses mentioned above, the addition of nitrogen does not always trigger population-wide changes in susceptibility across a panel of genotypes. Instead, genotypes can vary in both the magnitude and direction of changes in susceptibility following nitrogen treatment. These multi-directional effects could be due to another key element that is not present in abiotic stress models, which is the pathogen. It is the host/pathogen interaction that is impacted by nitrogen fertilization and not only the plant response. Nitrogen fertilization has an impact on pathogen effector expression [26]. This modification of the virulence of the pathogen may be isolate-dependent like in other pathosystems [75].

Understanding the underlying genetic control of NIS is difficult to determine due to a relatively weak effect of nitrogen (8%) and a relatively rare NIS phenotype in temperate japonica (14%). Moreover, the heritability of the NISI phenotype was relatively low (0.1). Traits measured under combined stress often have lower heritability, making QTL identification difficult [41]. Thus an elevated heritability of robustness is not easy to reach because it may not be related to a specific gene or genes of large or moderate effect, but instead due to many genes scattered throughout the genome contributing to a certain level of robustness to a stress [76]. Therefore, there might be other loci with modest effects on the phenotype not identify in this study. For example, in a robust genotype, the presence of functional redundancy between different genes could buffer the immune response to changes in the environment [76]. In that case, the resistance will be conferred by one gene in N0 environment and by the redundant gene in N1 environment.

The NIS phenotype is relatively rare (14%) in our panel. To understand why resistance in temperate japonica is so frequently robust to nitrogen fertilization, we searched for possible reasons why the *NIS3–3* allele is widespread in the temperate japonica sub-population. The *NIS*3 locus contains the *RIPER6* gene, with a high-frequency allelic variant conferring cold-resistance in the temperate japonica population [64]. Among the 3000 genomes available for screening, this allelic variant of RIPER6 was found in 90% of temperate japonica that had the detrimental *NIS3*–3 allele (Additional file 16). Thus in temperate japonica, alleles promoting robustness may be linked with allelic variants of genes conferring high fitness in a temperate environment [77]. This suggests that robustness of susceptibility to rice blast may have arisen through a hitchhiking effect from selection for cold hardiness.

While a lack of study does not yet allow a full understanding of the mechanisms involved in robustness of resistance in variable environments, several hypotheses can be proposed. A first family of hypothesis to explain the increase in susceptibility in the presence of nitrogen could be the existence of biotic stress regulators that may be down-regulated by nitrogen or possibly regulating the cross-talk between primary metabolism and the defense response. Consistent with the later mechanism, a GWA study using the fold-change in expression of PLANT DEFENSIN1.2 (PDF1.2) transcripts between combined and single hormonal treatments identified a locus involved in the cross-talk between biotic and abiotic stress [40]. Since some alleles of *NIS3* confer susceptibility per se, this locus could regulate blast susceptibility under nitrogen by such a mechanism. Indeed, molecular regulation at the transcriptional level is not necessarily the sum of the responses of each condition in a combined environment [78]. If an allele functions to allow the immune response to always exceed a sufficient threshold despite certain disturbances, this makes the final phenotype robust to those disturbances.

A second hypothesis is that NIS may be due to the presence of a regulator of primary metabolism targeted by *M. oryzae* to enhance its infection process. *NIS2* contains the gene *NADH-GOGAT2* encoding a glutamate synthetase, an enzyme active in leaf plastids that converts glutamine to glutamate [79]. The reverse reaction is catalyzed by Glutamine Synthetase (GS) allowing the production of Glutamin from Glutamate and NH4^+^ [80]. Several observations indicate that the NADH-GOGAT2 enzyme could be a good candidate for *NIS2*. Indeed, the GS/GOGAT cycle has already been shown to be a determinant in plant-pathogen interactions, impacting different plant defense strategies [71]. Moreover we have previously shown that the *GS1–2* gene was differentially expressed upon blast infection under high nitrogen conditions and is involved in NIS [26]. In addition, the rice *NIS1* locus that confers NIS contains the *NADH-GOGAT1* gene [24]. Finally, glutamate has been shown to induce resistance to *M. oryzae* after root application in rice [81] and in *A. thaliana* [82]. All these results suggest that the Glutamate/Glutamine cycle has a strong impact on the rice-blast pathosystem. The addition of extra nitrogen the day before inoculation could disrupt this cycle, causing NIS in some genotypes. Genotypes with the *NIS2–1* allele could have different NADH-GOGAT activities and thus be more sensitive to an input of nitrogen. Consistent with this hypothesis, the rice Kasalath genotype (from which *NIS1* susceptible allele was isolated) is known to be more susceptible to rice blast in the N1 condition and to display less NADH-GOGAT protein than Nipponbare, a genotype where nitrogen is known to have less of an impact on resistance [83].

## Conclusion

The **r**ice blast/japonica temperate rice pathosystem is partially impacted by NIS. We have adapted an index traditionally used for the study of abiotic stress to the study of NIS. This NIS index was used in a GWA study, in parallel with traditional methods using susceptibility phenotypes. Three new QTL were identified with different effects. First, the R allele of *RRobN1* locus provided resistance that was robust to the different nitrogen fertilization regimes, and should be selected in breeding programs. Second, the *NIS2–1* allele enhanced NIS, an effect potentially arising from the actions of a *NADH_GOGAT2* gene, and should be avoided in breeding programs. Third the *NIS3* locus, which was detected using the NIS index, confers susceptibility per se and enhances susceptibility under high nitrogen. The detrimental *NIS3–3* haplotype has possibly increased in frequency by genetic hitchhiking with an otherwise favorable allele for cold resistance, resulting in a reduced plasticity of susceptibility to nitrogen in the temperate japonica.

## Supplementary Information

**Additional file 1. ***List of varieties phenotyped with CD203 strains and used for GWAS.*

**Additional file 2. ***List of varieties phenotyped with CL26 strains.*

**Additional file 3. ***List of varieties used as validation set.* Varieties have been selected for their k-group classification of 19 SNPs selected to characterize the NIS3 QTL.

**Additional file 4. ***Disease severity after inoculation with CL26 strain depending on Nitrogen conditions.* Each point corresponds to the Lsmean of number of rice blast lesion for one genotype. In white, N0 corresponds to the low nitrogen condition, in grey N1 corresponds to the high nitrogen condition. Red diamonds corresponds to Lsmean for each treatment. The strains used for inoculation is CL26.

**Additional file 5. ***Analysis of Variance table of Nitrogen and Genotype effect on the number of lesion of rice blast by cm*^*2*^ *of leaves.* ANOVA calculated from linear model. Significance level at 0.05.

**Additional file 6. ***Descriptive Statistic of Rice blast susceptibility by level of Nitrogen and NISI on the number of lesion of rice blast by cm*^*2*^ *of leaves.*

**Additional file 7. ***Distribution of Nitrogen Induced Susceptibility Index.* 50% of panel has a NIS Index between − 2 and 2.

**Additional file 8. ***Significant SNPs detected in each GWAS.* A SNP is considerate as significant when -log(P) ≥ 5.

**Additional file 9. ***QTLs detected from GWAS of Blast Susceptibility with nitrogen interaction.* Each QTL is given with the traits that led to its identification, the number of significant (−log(P) ≥ 5) and sub-significant (4 ≤−log(P) < 5) SNPs contained, the maximum LOD score of the pics associated, and the number of genes present in the region.

**Additional file 10. ***RRobN and NIS2 effect on Number of lesions of CL26 strains infection.* Each point corresponds to the number of rice blast lesion on one leaf. N0 corresponds to the low nitrogen condition, and N1 is the high nitrogen condition. Red Diamond correspond to the LSmean of each allele of each QTL. Groups from a pairwise comparison with an independent Tukey adjustment for each QTL based on a model with Trials and genotype as covariates. The strains used is CL26.

**Additional file 11. ***Genes list present in RRobN QTL*. Gene functions from FunRiceGenes database. Thirteen unidentified genes, five retrotransposon genes, and two transposons have been omitted of this list.

**Additional file 12. ***Genes list present in NIS2 QTL*. Genes function was annotated from FunRiceGenes data base and Orygenes database. Nineteen unidentified genes, three retrotransposon genes, and three transposons have been omitted from this list.

**Additional file 13. ***2 major haplotypes for NIS3 present in the sample susceptible to CD203 determinate by k-group calculation with rice 3000 genomes database references.*

**Additional file 14. ***Adjusted NIS Index by NIS3 allele with CL26 M.o strains infection.* Each point correspond to the adjusted *NIS index* of one repetition of one genotype calculated as: $$ 1-\raisebox{1ex}{$ LSmeanN1$}\!\left/ \!\raisebox{-1ex}{$ LSmeanN0$}\right. $$. *P*-values are from a wilcoxon test.

**Additional file 15. ***Genes list present in NIS3 QTL with* Gene function from FunRiceGenes data base and Orygenes database. The fifth column correspond to the gene differentially expressed in Huang et al. 2017*.* Nineteen unidentified genes, three retrotransposon genes, and three transposons have been omitted from this list.

**Additional file16.** Frequency of NIS3 haplotype by subpopulation in 3000 genomes.

## Data Availability

phenotypic data are available on demand. Seeds can be obtained from Biological resource center for seeds (BRC GAMèT) in Montpellier.

## Abbreviations

NIS: Nitrogen Induced Susceptibility
NISI: Nitrogen Induced Susceptibility Index
QTL: Quantitative trait locus
GWAS: Genome wide association study
N0: Low nitrogen condition
N1: High nitrogen condition
LSMeans: Last Square Means
NI: Nitrogen Impact
h2: Heritability
SNP: Single nucleotide polymorphism
LD: Linkage disequilibrium
PCA: Principal Component Analysis
MLM: Mixed linear model
