# Supplementary figures and images for: Genome-wide association of rice response to blast fungus identifies loci for robust resistance under high nitrogen
Source: BMC Plant Biol. 2021 Feb 18;21:99. doi: 10.1186/s12870-021-02864-3 (PMC7893971; doi:10.1186/s12870-021-02864-3)

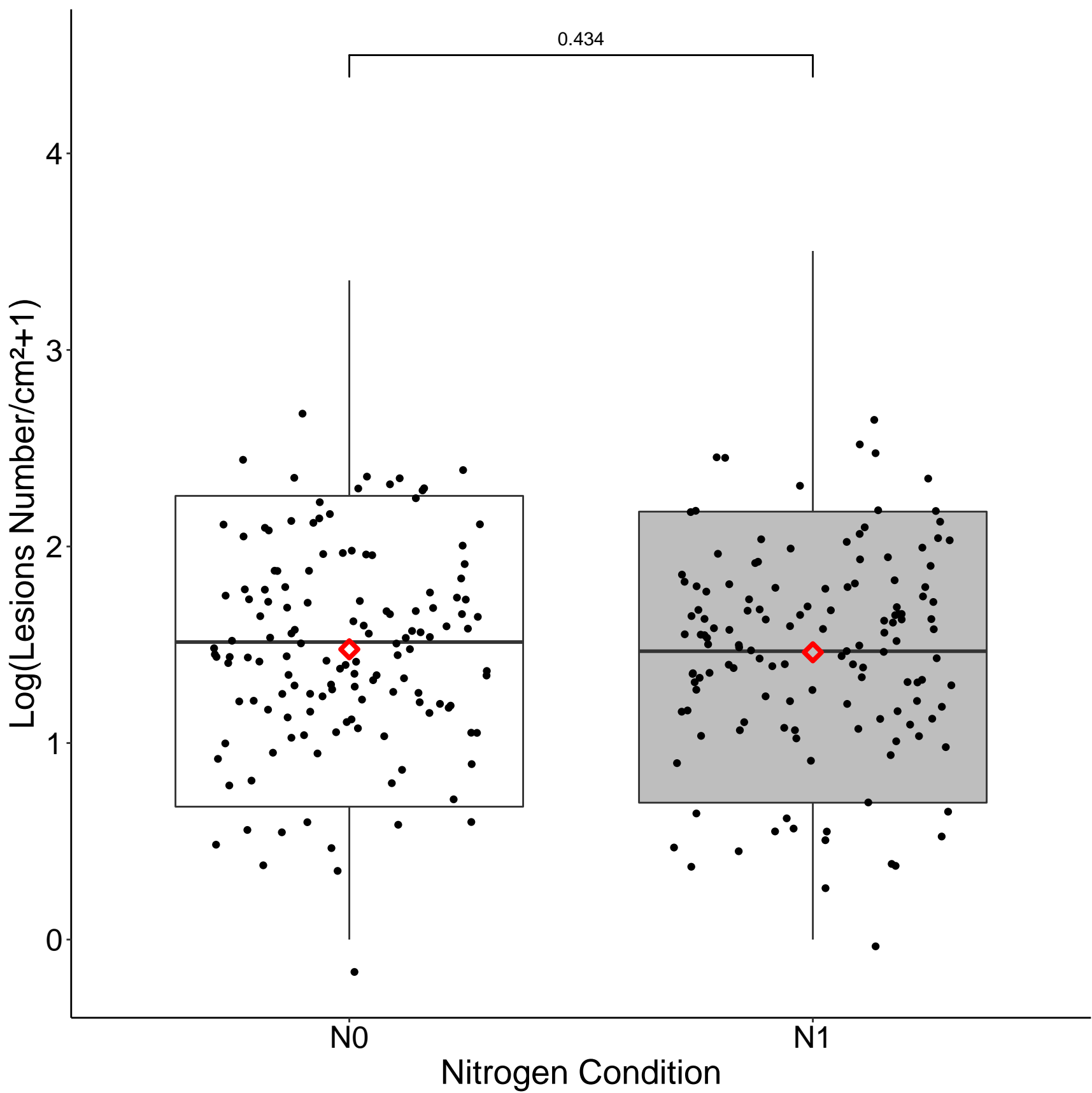

Supplement: Supplementary file 4 — Additional file 4. Disease severity after inoculation with CL26 strain depending on Nitrogen conditions. Each point corresponds to the Lsmean of number of rice blast lesion for one genotype. In white, N0 corresponds to the low nitrogen condition, in grey N1 corresponds to the high nitrogen condition. Red diamonds corresponds to Lsmean for each treatment. The strains used for inoculation is CL26. [file 12870_2021_2864_MOESM4_ESM.pdf]

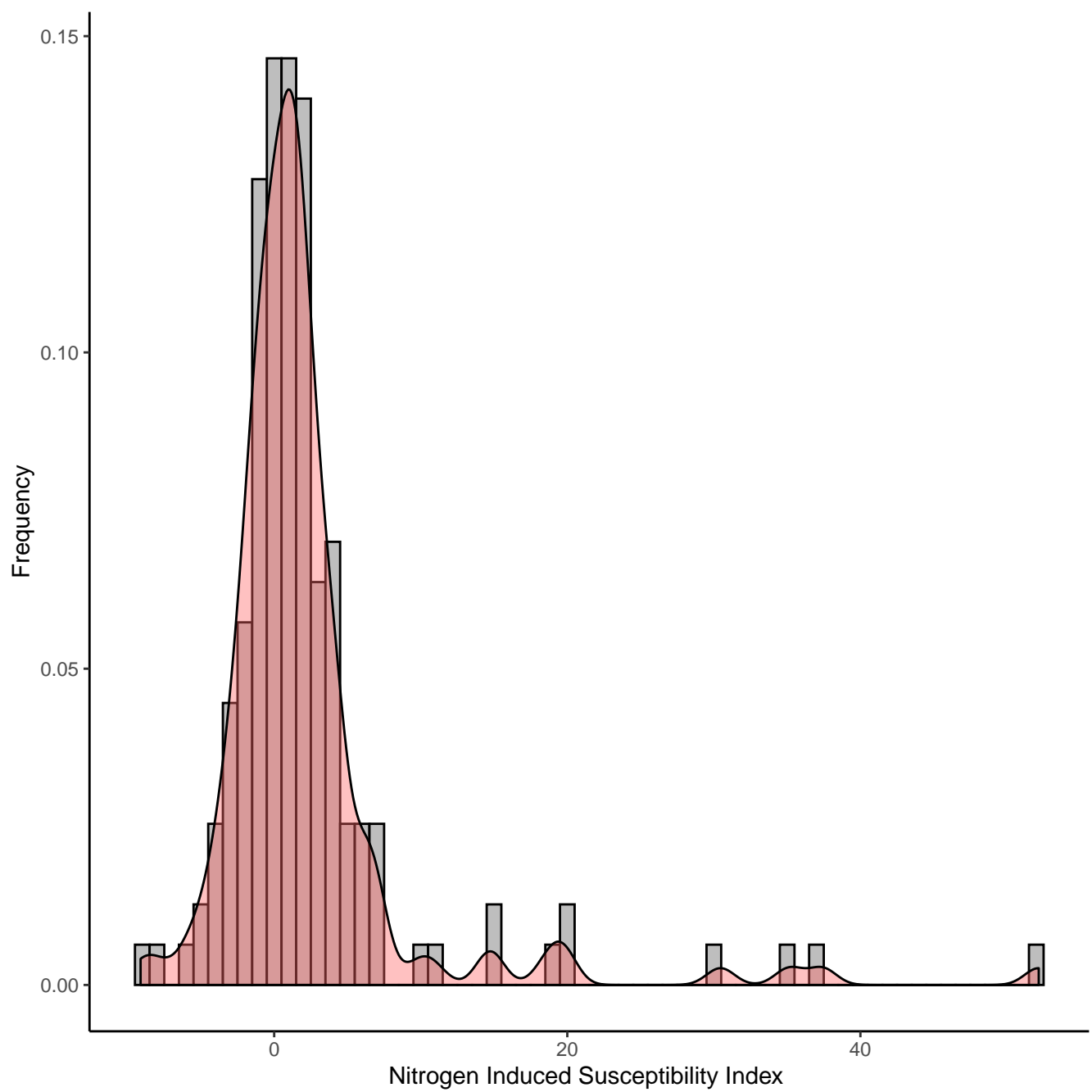

Supplement: Supplementary file 7 — Additional file 7. Distribution of Nitrogen Induced Susceptibility Index. 50% of panel has a NIS Index between − 2 and 2. [file 12870_2021_2864_MOESM7_ESM.pdf]

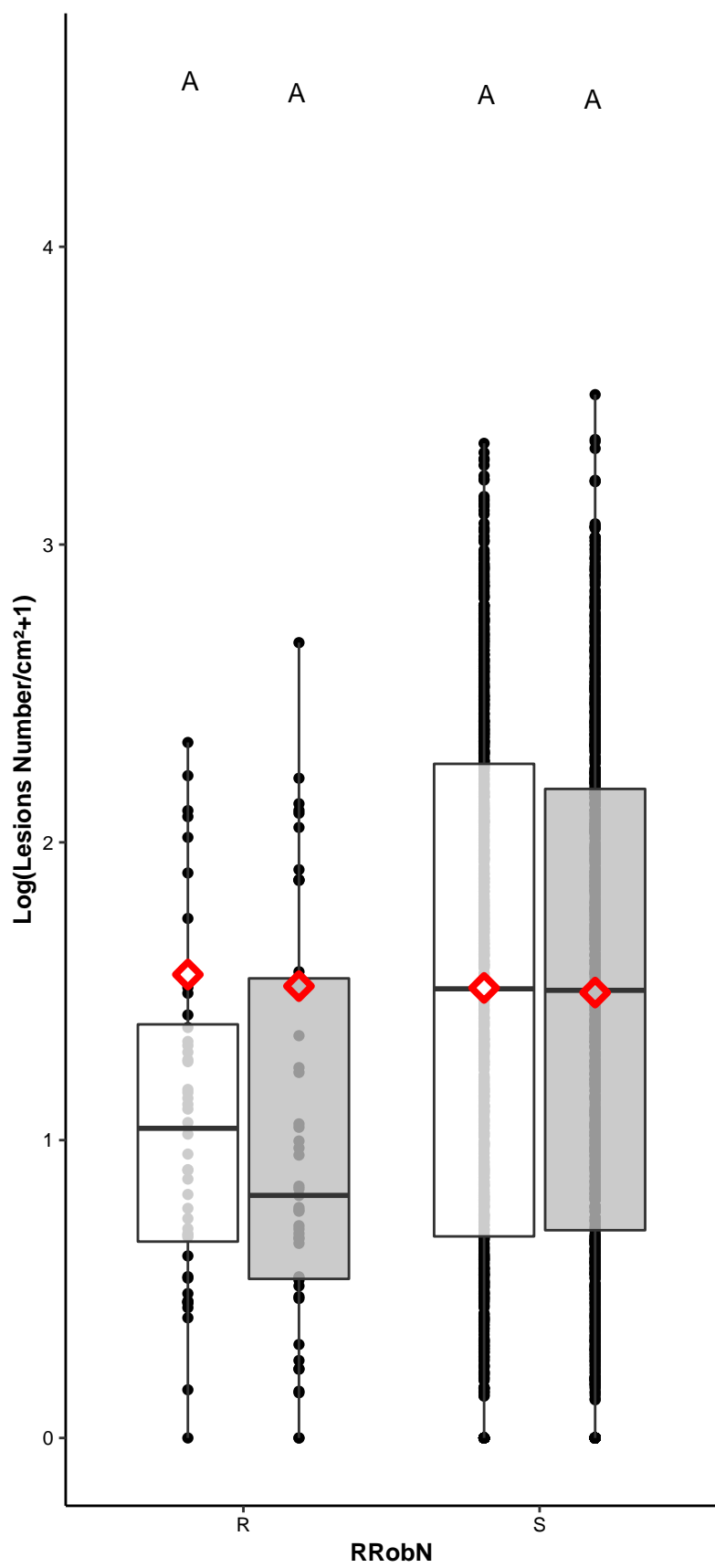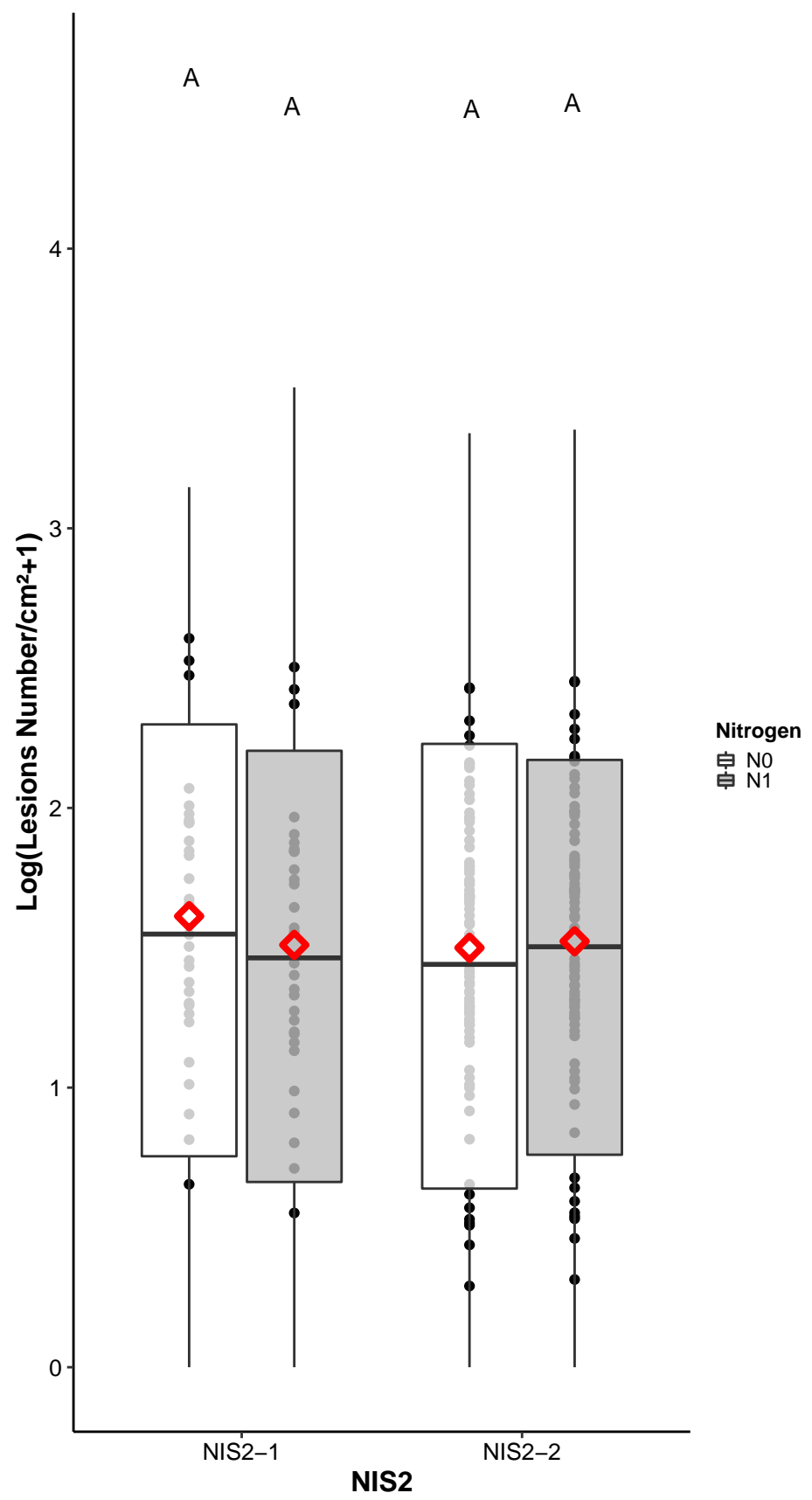

Supplement: Supplementary file 10 — Additional file 10. RRobN and NIS2 effect on Number of lesions of CL26 strains infection. Each point corresponds to the number of rice blast lesion on one leaf. N0 corresponds to the low nitrogen condition, and N1 is the high nitrogen condition. Red Diamond correspond to the LSmean of each allele of each QTL. Groups from a pairwise comparison with an independent Tukey adjustment for each QTL based on a model with Trials and genotype as covariates. The strains used is CL26. [file 12870_2021_2864_MOESM10_ESM.pdf]

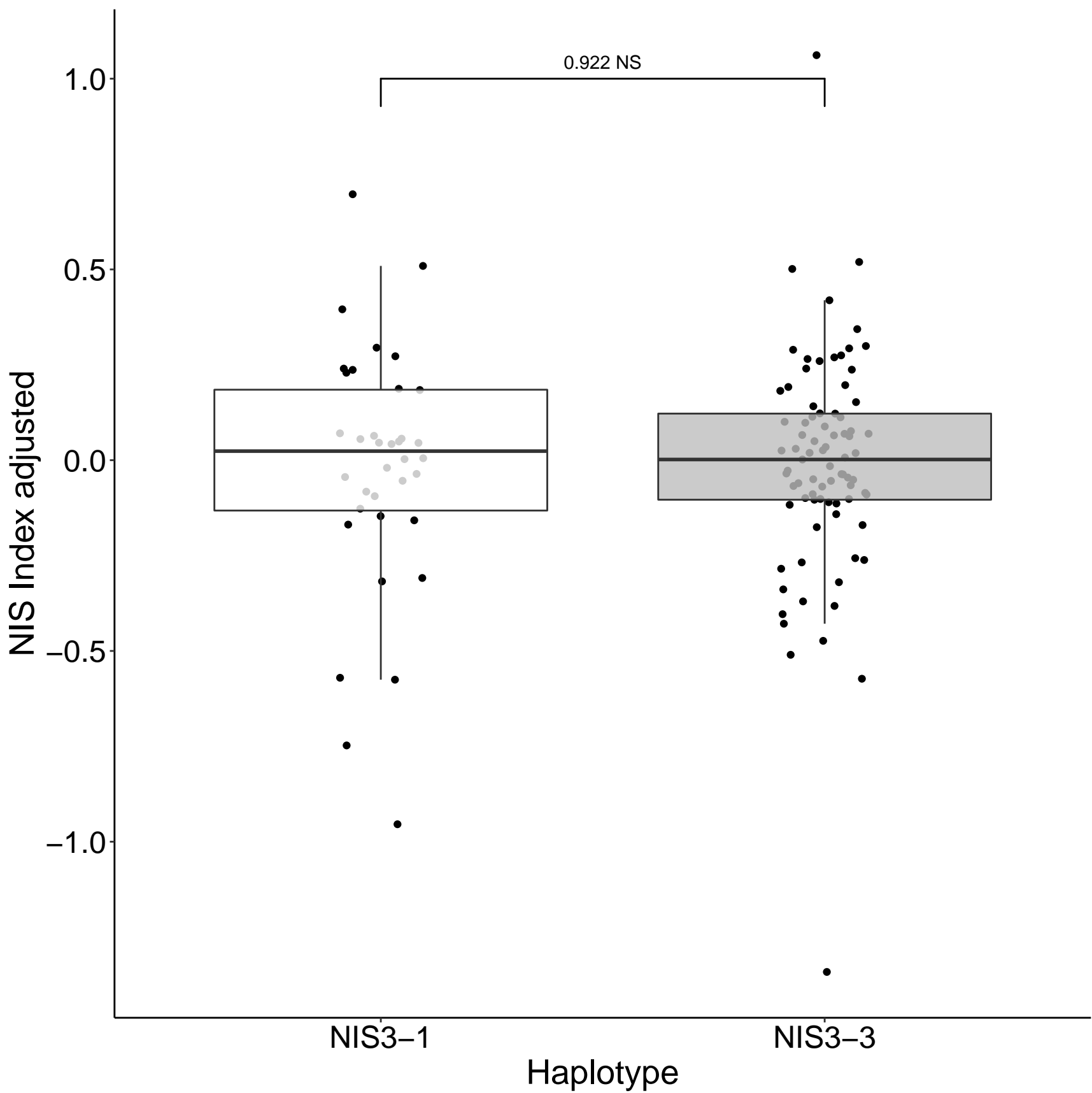

Supplement: Supplementary file 14 — Additional file 14. Adjusted NIS Index by NIS3 allele with CL26 M.o strains infection. Each point correspond to the adjusted NIS index of one repetition of one genotype calculated as: $$ 1-\raisebox{1ex}{$ LSmeanN1$}\!\left/ \!\raisebox{-1ex}{$ LSmeanN0$}\right. $$1−LSmeanN1LSmeanN0. P-values are from a wilcoxon test. [file 12870_2021_2864_MOESM14_ESM.pdf]
